# DNA-PRESENT: An Improved Security and Low-Latency, Lightweight Cryptographic Solution for IoT

**DOI:** 10.3390/s24247900

**Published:** 2024-12-11

**Authors:** Maria Imdad, Adnan Fazil, Sofia Najwa Binti Ramli, Jihyoung Ryu, Hairulnizam Bin Mahdin, Zahid Manzoor

**Affiliations:** 1 Department of Avionics Engineering, Air University, E-9, Islamabad 44000, Pakistan; drmariaimdad@gmail.com; 2 Faculty of Computer Science and Information Technology, Universiti Tun Hussein Onn Malaysia, Parit Raja, Batu Pahat 86400, Malaysia; 3 Electronics and Telecommunications Research Institute (ETRI), Gwangju 61012, Republic of Korea; 4 Department of P & M, PIR Mehr Ali Shah Arid Agriculture University, Rawalpindi 46000, Pakistan

**Keywords:** diffusion property, DNA cryptography, Internet of Things (IoT), lightweight cryptography, performance analysis, PRESENT block cipher, security analysis

## Abstract

The vast interconnection of resource-constrained devices and the immense amount of data exchange in the Internet of Things (IoT) environment resulted in the resurgence of various security threats. This resource-constrained environment of IoT makes data security a very challenging task. Recent trends in integrating lightweight cryptographic algorithms have significantly improved data security in the IoT without affecting performance. The PRESENT block cipher, a standard and lightweight benchmark algorithm, is a widely accepted and implemented algorithm with a simple design, low-cost implementation, and optimum performance. However, this simple design utilizing lightweight linear and non-linear functions led to slow confusion and diffusion properties. The static bits in the permutation layer are the leading cause of slow diffusion, showcasing dependencies between plaintext and ciphertext bits. This research addresses and seeks to overcome this shortcoming of slow confusion and diffusion using the Deoxyribonucleic Acid (DNA) replication process and shift-aided operations, leading to the DNA-PRESENT block cipher. Security, cost, and performance analyses were performed to verify the improvements. The results demonstrated that with only 33.5% additional cost, DNA-PRESENT increased key sensitivity to 73.57%, plaintext sensitivity to 33%, and consistently ensured an average bit error rate (BER) of 50.2%. An evident increase of 176.47 kb/s in throughput and reduced latency to 17 cycles/block kept the good hardware efficiency of 43.41 kbps/KGE, and the reduction in execution time by 0.2333 s led to better performance. Considering the security advances achieved, this cost increase is a trade-off between security and performance.

## 1. Introduction

The recent advancements in internet architectures [1] and their extended integration with resource-constrained devices have facilitated continuous communication, resulting in ever-increasing data generation [2] in IoT networks. Device deployment at remote locations with little to no physical security [3] unlocks the susceptibility to data theft, access control violations, and network attacks [4,5]. The devices, ranging from sensors, actuators, and radio frequency identification (RFID) tags, are responsible for data transmission and reception in a confidential manner [6,7]. Lightweight cryptographic (LWC) algorithms were designed to address the growing demand for data confidentiality in IoT networks [6].

**Figure 1:**
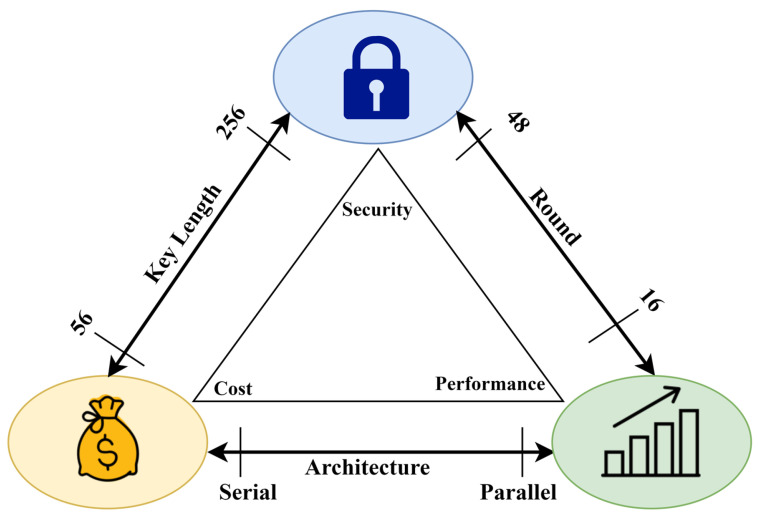
Cost, security, and performance trade-off triangle [10].

The design of an LWC algorithm requires a perfect balance between cost, security, and performance in the architectural design triangle, as presented in Figure 1 [8,9,10,11]. However, the algorithm must not exceed the gate limit of 3000 GEs for security implementation [12,13,14]. Hence, simple mathematical functions—especially substitution, permutation, shift, and XoR—are prioritized to achieve an optimal balance between low cost, good security, and better performance [12]. The block ciphers in LWC have a Substitution Permutation Network (SPN) structure characterized by alternating layers of substitution box (s-box) and permutation (p-box). This design ensures confusion and diffusion within the algorithm. Confusion obscures the statistical relationship between keys and ciphertext, while diffusion conceals a similar relationship between plaintext and ciphertext [15,16,17,18,19,20,21,22].

In recent years, numerous lightweight block cipher solutions have been considered for IoT applications, including AES [13]; NOEKEON [23]; ICEBERG [24]; mCrypton [12]; PRESENT [25]; PUFFIN [12,26]; LED [12,27]; PRINCE [12]; RECTANGLE [28]; and I-PRESENT [29]. The PRESENT block cipher is an LWC standard in the International Organization for Standardization (ISO)/International Electrotechnical Commission (IEC) 29192. A simple, yet efficient design of the PRESENT block cipher is still considered a benchmark for the design of new lightweight algorithms, as it ensures an effective balance between cost and performance [30]. The present block cipher has an SPN structure, with wide applicability and acceptance in IoT applications [25,27,31,32,33]. Despite its wide acceptance and adoption, the algorithm’s design needs security improvements [22,34,35,36].

Recent cryptanalysis advancements have led to the exploitation of any specific part of the algorithm instead of attacking the whole algorithm [22,37]. An in-depth study of PRESENT from the literature establishes two basic weaknesses in PRESENT, which need to be addressed. First, refs. [31,38,39,40] PRESENT has a weak key schedule with evident dependency between round keys, resulting in a slow and predictable bits transition. This issue has been addressed using an improved KSA, using linear and non-linear functions, ensuring improved confusion [41].

**Figure 2:**
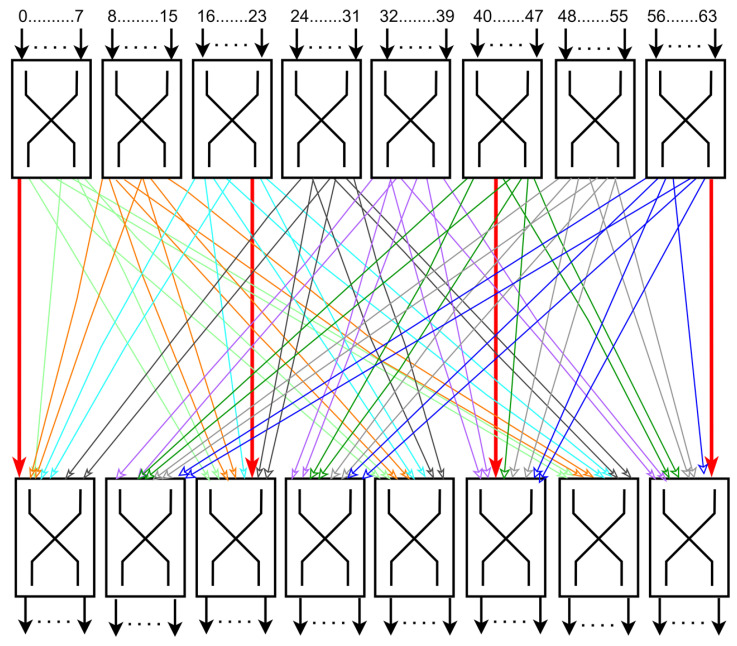
Hourglass design of the *pLayer()* [44].

Secondly, the produced ciphertext also has low diffusion properties due to the simple design of the permutation box (pLayer) and the presence of non-permuted static bits at multiple locations [42,43,44]. The design of PRESENT’s pLayer is kept simple to limit hardware cost, but the leftmost bit (bit 0), central bits 21 and 42, and the rightmost bit (bit 63) are never permuted—these bits are static. This permutation box design is an hourglass design, as explained in [44] and presented in Figure 2, keeping the leftmost, central, and rightmost bits unaltered. This is one of the leading causes of slow diffusion in ciphertext, endorsing that the permutation layer alone cannot introduce enough diffusion [44,45,46] and requires the identification of other methods to enhance the diffusion property of the algorithm.

Several efforts have been made to address the slow diffusion and the corresponding improvements in the PRESENT block cipher. The most relevant and impactful of these are discussed here. In the year 2016, an improved PRESENT using a dynamic s-box was presented [45]. The improved design was compared against PRESENT for the avalanche effect, where significant security improvements regarding the diffusion property were observed in the improved design. In the year 2020 [46], a new variant, D-PRESENT, was introduced, which used dynamic key-dependent s-boxes to improve the randomness in the ciphertext. D-PRESENT successfully increased the avalanche effect and reduced the number of rounds to half, which resulted in execution time reduction. However, calculating the active s-box before each iteration can significantly increase the algorithm’s complexity. In the year 2021 [44], the authors improved the SPN structure using the keyed permutation of PRESENT to ensure resistance against side-channel attacks that resulted in different bit permutations for each round. However, a thorough analysis showed that this technique also increased system-level overhead and power consumption. Recent research by Ali Abdelli et al. [46] improved the security of the PRESENT block cipher using chaotic systems to achieve robustness and better randomness for resource-constrained devices. All these approaches mentioned above improved the PRESENT block cipher using recent cryptographic advancements. However, there has been no evidence where the cumulative impact of the improvements on security, cost, and performance has been studied collectively. Moreover, this suggests that the algorithm’s weakness should be mitigated through an alternative low-cost diffusion technique.

With the recent advances in cryptography, many researchers have used DNA cryptography as a means to enhance the security of encryption algorithms [47,48,49] because the effectiveness of any DNA-based genetic scheme is directly associated with the level of randomness it offers [50,51]. In addition to providing randomness, DNA cryptography ensures robust confusion and diffusion, and fast and low-cost implementation functions [52,53,54,55]. Building on the research mentioned earlier, the diffusion property of the algorithms can be enhanced using operations derived from Deoxyribonucleic Acid (DNA) cryptography. Another research [14] used a DNA-based transposition layer and randomly chosen DNA sequences for the generation of secret keys, resulting in additional image security. Recent research used DNA-sequencing and permutation-based functions to ensure image security with improved entropy, reduced time, and complexity [55]. The DNA-based symmetric cryptographic algorithm in [56] partitions encryption data into blocks and employs a symmetric secret key that is directly extracted from a set of chromosomes; its implementation on Raspberry Pi’s platform indicated low power consumption and reduced algorithmic complexity. The Chinese remainder theorem was utilized to construct a knapsack algorithm by Satpati [57], as the method is a public-key encryption system and the dissemination is slower than private-key encryption. They improved the performance by employing DNA encoding along with the Rail Fence, resulting in additional expected security.

**Figure 3:**
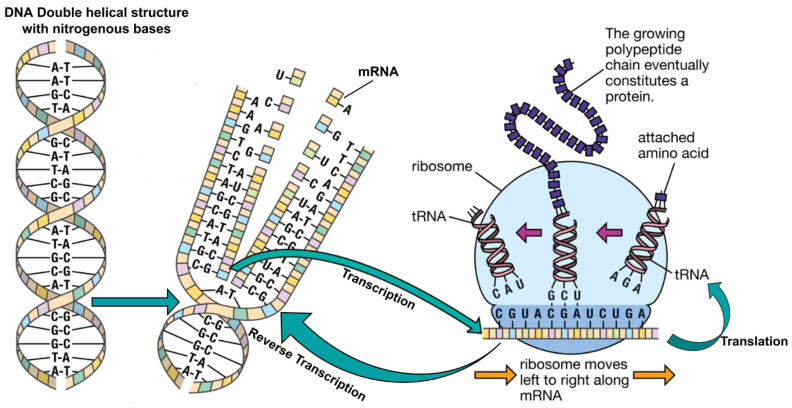
Biological DNA replication process.

In the year 2021 [18], Thabit introduced a two-layer encryption scheme based on genetics and DNA operations. The researcher explained that the randomness in the nucleotide bases can be efficiently applied to create reliable yet robust cryptographic schemes. The two-layer architecture efficiently integrates the DNA properties, with a first traditional layer of Feistel and SPN structure designed to offer Shannon’s confusion and diffusion properties using conventional operations such as XOR, XNOR, and shift. The second layer was inspired by the Central Dogma of Molecular Biology (CDMB), using DNA replication as an additive low-cost diffusion solution. The DNA replication process simulates the natural process of genetics. The mathematical simulation of this natural process has the following steps—DNA coding, Transcription, Translation, and Reverse Transcription—depicting a complete biological replication process, as presented in Figure 3. The proposed algorithm was evaluated on its throughput and execution time to validate the expected improvements. Moreover, the authors also compared the proposed algorithm against other state-of-the-art algorithms to prove that fast processing and improved security were achieved. All these abovementioned researches highlight that the inherent diffusion-based shortcomings of cryptographic solutions can be addressed using DNA cryptography. Moreover, a thorough evaluation of security, cost, and performance is necessary to assess the impact of these improvements on the overall effectiveness of the system, as presented in Figure 1.

In this article, we propose the integration of CDMB-inspired DNA replication, along with left–right shifts, in the PRESENT block cipher as a low-cost diffusion solution, called DNA-PRESENT. This enhancement increases the confusion and diffusion properties while keeping the block and key size unchanged. DNA-PRESENT was thoroughly evaluated and the obtained results from the avalanche effect, correlation coefficient, and bit error rate tests prove the improved diffusion and security, where hardware evaluation endorses better performance with increased cost.

This study differentiates itself from past efforts to improve the PRESENT block cipher in two major respects. First, it introduces a DNA-based replication approach to facilitate low-cost confusion and diffusion, a novel method not previously investigated. Although other methods have been used, they come with their own limitations, as highlighted earlier. This research also extensively tests the algorithm, covering parameters such as security, cost, and performance, thereby addressing all three components of the LWC evaluation triangle (Figure 1). Unlike previous enhancements, which are often assessed based on a single property, this study’s methodology provides a robust framework that could be used as an evaluation standard for future LWC algorithms as well.

The paper’s organization follows the current section as an introduction. Section 2 provides a detailed description of the PRESENT block cipher. Section 3 discusses the design details of the proposed DNA-PRESENT and the design decisions. Section 4 presents the security cost, performance analysis, and a detailed discussion highlighting the research findings. Section 5 outlines a differential cryptanalysis approach of DNA-PRESENT to find the optimal number of rounds. Section 6 presents the comparitive analysis of the proposed scheme with the relevant benchmarks. Finally, Section 7concludes the study by presenting a summary and critique of the findings.

## 2. PRESENT Encryption

The PRESENT block cipher has two main parts: round key generation using *generateRoundKeys()* and data encryption. Round keys are generated on the fly and subsequently used in each round. The *generateRoundKeys()* function generates 31 different round keys for each round *“i”* using the provided single secret key. Here, *“i”* is the round number and its value ranges between i≤32. However, in the first encryption round, the round key is extracted directly from the secret key, followed by the use of 31 round keys from *generateRoundKeys()*. The PRESENT block cipher has 64 bits of plaintext, which are referred to as STATE, $\left[S_{63},S_{62},S_{61}\ldots \ldots S_{0}\right]$.

**Figure 4:**
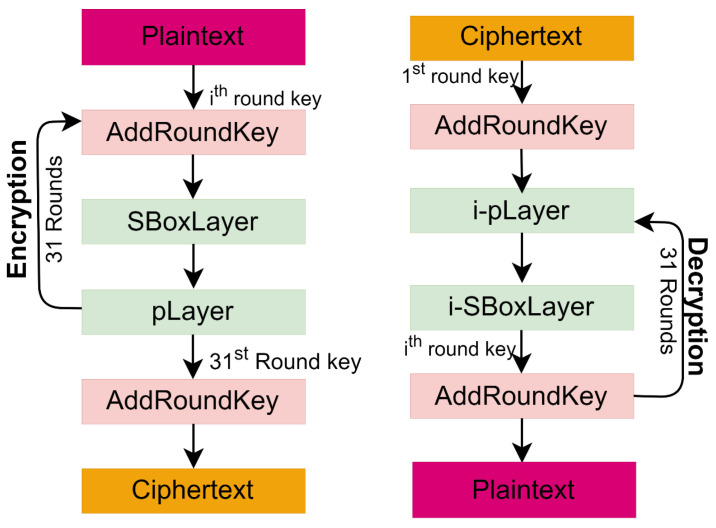
Encryption/decryption in PRESENT block cipher.

The encryption begins by performing an XoR operation between STATE and the first-round key in *AddRoundKey()*. This step is succeeded by the application of nonlinear layer *sBoxLayer()*, a substitution layer. Subsequently, a linear permutation layer *pLayer()* is applied to complete the first round of encryption. This process is repeated for 31 rounds, and after this, the output of the 31st round undergoes an additional key whitening round, generating the ciphertext using the last round key. The pseudocode of the encryption process is presented below in C language as Algorithm 1. The complete encryption and decryption process of the PRESENT block cipher is presented in Figure 4, depicting that the decryption is the exact reversal of the encryption process.
**Algorithm 1.** Pseudocode of PRESENT block cipher Encryption1: *generateRoundKeys()*2: **for** *i* = 1 to 31 **do**     3: *addRoundKey*(STATE,$K_{i}$)     4: *sBoxLayer*(STATE)     5: *pLayer*(STATE)6: **end for**7: *addRoundKey*(STATE, $K_{31}$)

### 2.1. AddRoundKey()

**Figure 5:**
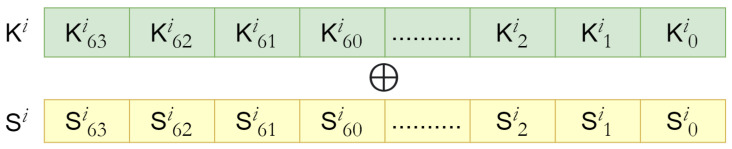
*AddRoundKey()* in PRESENT block cipher.

In *AddRoundKey()*, each bit of the 64-bit STATE is XoRed with a corresponding round key, as presented in Figure 5. In each round, the *AddRoundKey()* is followed by *sBoxLayer()* and *pLayer()*. However, the ciphertext is produced in the key whitening round, where only $K_{31}$ is XORed with STATE presented in Algorithm 1.

### 2.2. SBoxLayer()

*SBoxLayer()* (Substitution box) is the nonlinear layer; it is a 4-bit by 4-bit substitution $S:F_{2}^{4}$ to $F_{2}^{4}$. Its hexadecimal representation is given in Table 1: X is input, and S(X) is substituted bits. The STATE from *AddRoundKey()* is subjected to *SBoxLayer()* by substituting each bit using the substitution box presented in Table 1.

### 2.3. pLayer()

**Figure 6:**
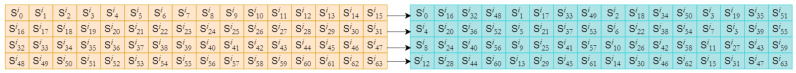
*pLayer()* in PRESENT block cipher.

A *pLayer* is a permutation box, where a bit located at position *“i”* of the STATE is relocated to position *P(i)*, as specified by Equation (1) [58]. This simple permutation process is presented in Figure 6.

**Figure 7:**
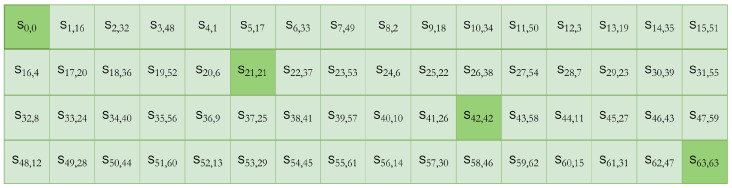
Cipher state of PRESENT block cipher using *pLayer()*.

The plaintext that completed one round of encryption through *addRoundKey()*, *SBoxLayer()*, and *pLayer()* is referred to as the cipher STATE, which is the input for the next round. Figure 7 represents the cipher state at any given round *“i”*, showing that the bits at positions 0, 21, 42, and 63 remain unchanged and are never permuted, leading to slow and predictable diffusion after each round. This *pLayer()* design is known as the hourglass design, shown in Figure 2 [44], indicating the fixed bits of the hourglass. This requires improving the *pLayer()* of a PRESENT block cipher for, ultimately, more robust diffusion.

(1) $$P\left(i\right)=\left\{\begin{array}{cc} 16\times i\;mod\;63 & for\;0\leq i\leq 63.\\ 63 & for\;i=63. \end{array}\right.$$

## 3. Proposed DNA-PRESENT Block Cipher

**Figure 8:**
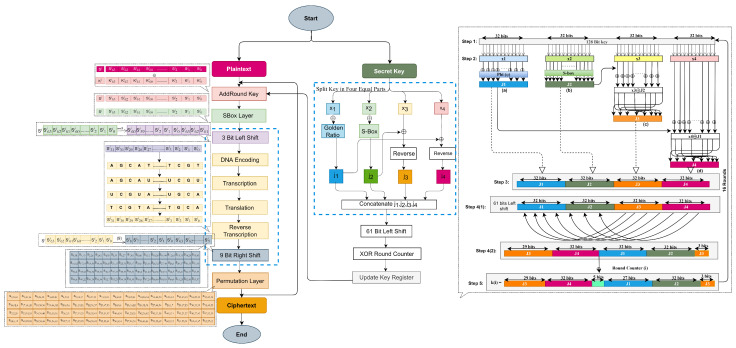
A layer-by-layer descriptive diagram of DNA-PRESENT.

This section describes the key contribution of this research, which is the incorporation of three new layers to increase the diffusion of the PRESENT block cipher. These layers are the left shift, DNA replication, and right shift [21,44,59], formally introducing DNA-PRESENT. The key schedule algorithm was improved recently, as presented in [41], and is integrated here as it augments the security of the PRESENT block cipher. The descriptive diagram with new diffusion layers and improved KSA from [41] is presented in Figure 8, demonstrating the full architecture and specifics of each layer within DNA-PRESENT. Given below is the detailed design of the proposed DNA-PRESENT block cipher.

### 3.1. DNA-PRESENT Encryption Process

**Figure 9:**
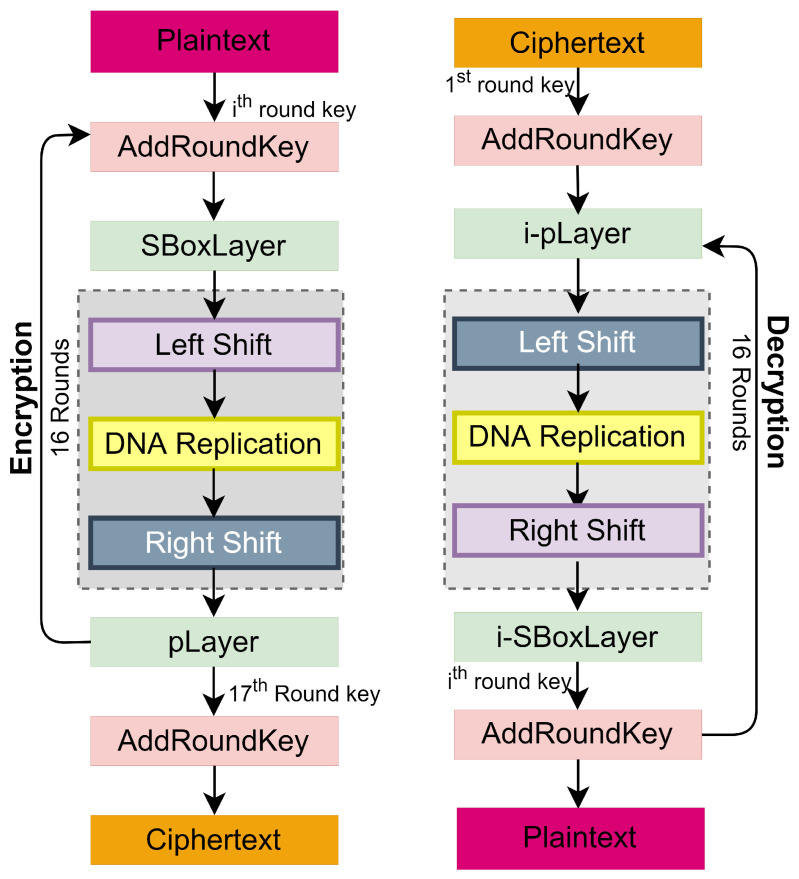
Encryption and decryption of DNA-PRESENT block cipher.

The PRESENT block cipher’s SPN structure has been improved, with three additional layers aimed at increasing ciphertext diffusion. The challenge while adopting any diffusion layer is that it may slow the encryption process. However, the selected layer has already been used as a low-cost diffusion [38], and the shift operation will break the sequence of the cipher state. Moreover, it has been validated with gate requirements as a cost estimation. *generateRoundKeys()* generates round keys using the enhanced key schedule presented and validated in [41]. The block diagram illustrating the encryption process is shown in Figure 9, and the pseudocode of encryption of DNA-PRESENT is given below, followed by the explanation of newly introduced diffusion layers. The encryption–decryption process of DNA-PRESENT is presented in Figure 9 and Algorithm 2, in which a total of 17 round keys are to be used, where 16 round keys are generated using the secret key and the secret key itself is the first round key. A similar process is repeated for decryption but in reverse order. In PRESENT and DNA-PRESENT, round keys are produced ahead of the encryption and decryption processes, with the sole difference being their utilization in reverse order.
**Algorithm 2.** Pseudocode of PRESENT block cipher Encryption1: *generateRoundKeys()*2: **for** *i* = 1 to 16 **do**     3: *addRoundKey*(STATE,$K_{i}$)     4: *sBoxLayer*(STATE)     5: *LeftShift*(STATE)     6: *DNAReplication*(STATE)     7: *RightShift*(STATE)     8: *pLayer*(STATE)9: **end for**10: *addRoundKey*(STATE,$K_{17}$)

**Figure 10:**
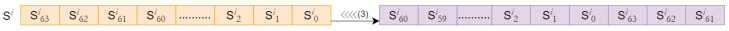
Left shift of STATE in DNA-PRESENT.

**Figure 11:**
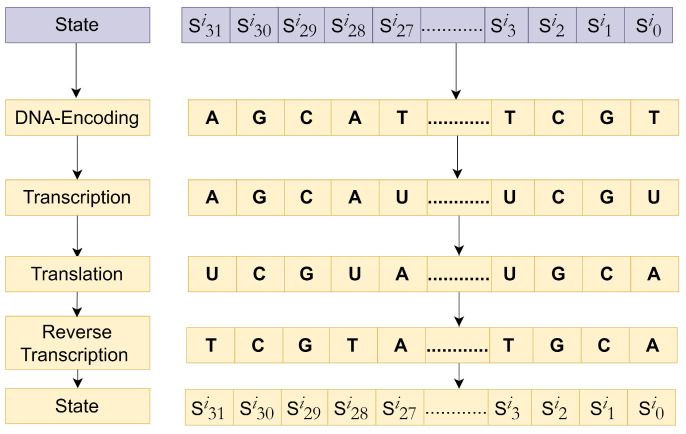
Step-by-step DNA replication process.

**Figure 12:**
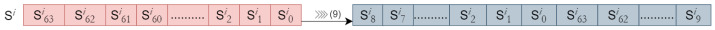
The right shift of STATE in DNA-PRESENT.

**Figure 13:**
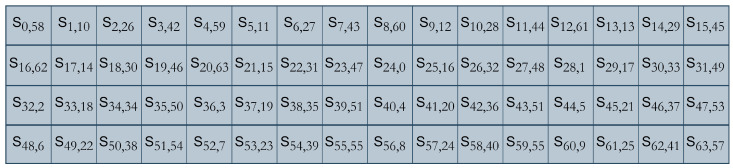
The cipher state after introducing three new layers in DNA-PRESENT.

1. **LeftShift()**: The STATE is the current value of the text, and at iteration *“i”*, this value is updated by a left shift of 3 bits, STATE = $\left[S_{63}\ldots S_{0}\right]$→$\left[S_{60},S_{59},\ldots \ldots S_{0},\ldots S_{61}\right]$, as in Figure 10.
2. **DNAReplication()**: DNA is a unique entity in living organisms, and its replication process (from Figure 3) has been simulated to enhance the randomness in PRESENT cipher [18]. Four bases in DNA make up the entire human protein structure—Adenine (A), Cytosine (C), Guanine (G), and Thymine (T). The following basic steps are involved in the DNA replication process presented in Figure 11.
  - The STATE is fragmented in two halves each comprising 32 bits, as presented in Figure 11. The left half of 32 bits is $\left[S_{31},S_{30},S_{29}\ldots \ldots S_{0}\right]$, and the right half is $\left[S_{63},S_{62},S_{61}\ldots \ldots S_{3}0\right]$. Following the natural DNA replication process in Figure 3, the new strand is built against the old strand. Hence, the left half stays unchanged; however, the right half undergoes replication.
  - Binary bits of the right half are converted into DNA base sequence using 00-A, 11-T, 10-C, and 01-G coding [60,61].
[11000110]DNA encoding[TAGC]
  - The DNA sequence undergoes conversion to mRNA by substituting the base Thymine (T) with Uracil (U); this process is referred to as *“Transcription”*.
[TAGC]mRNA sequence[UAGC]
  - In the next step, mRNA is transformed into a tRNA sequence using the DNA complement rule for bases, carrying a conversion of bases as A-U, U-A, G-C, and C-G, which is called the Biological *“Translation”* Process.
[UAGC]tRNA sequence[AUCG]
  - *“Reverse Transcription”* is applied on the obtained sequence where the Uracil base (U) is replaced with the Thymine (T) base.
[AUCG]Reverse transcription[ATCG]
  - After DNA replication, the sequence is converted into binary using A-00, T-11, C-10, and G-01 and combined with the original left half to create the updated STATE = $\left[S_{31},S_{30},S_{29}\ldots \ldots S_{2},S_{1},S_{0}\right]$.
3. **RightShift()**: The STATE obtained from the DNA replication layer is subjected to a right shift of nine bits, STATE = $\left[S_{63},\ldots \ldots S_{0}\right]$→$\left[S_{8},S_{7}\ldots S_{0},S_{63},\ldots \ldots S_{9}\right]$, as presented in Figure 12. These newly introduced diffusion layers have an evident impact on the dispersion of the binary bits. The previous bits that were fixed (using pLayer) and did not undergo any transition (as in Figure 7) have been dispersed, and a resultant matrix, after these newly introduced three layers result in cipher STATE, is presented in Figure 13.

### 3.2. The Design Rationale

**Figure 14:**
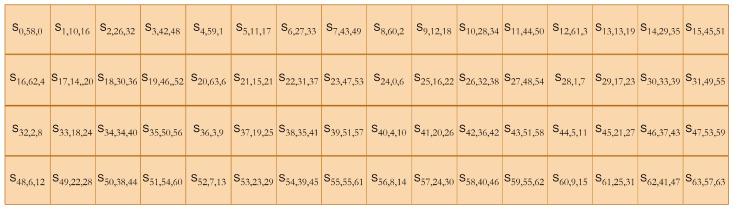
The cipher state after permutation layers.

DNA cryptography has served as a robust and fast technique in different scenarios while improving the security of modern cryptosystems [21,44,47,48,49,50,51,56,57,59,62]. The core of the diffusion improvement concept resides in the monitored cost and performance of the system. Diffusion is achieved at the minimum possible cost of implementation, particularly from the hardware perspective. This section offers an in-depth perspective on the design rationale while articulating the justifications for the design decisions implemented. The importance of each layer is given as follows:

- Left shift of three bits breaks the original sequence of the bits received from *SBoxLayer()*, and removing these three bits changes the sequence as a whole. It is evident from the hexadecimal notation that the entire sequence has changed. For example, if at any round the value of STATE from *SBoxLayer()* is “B3AE7609FAFF1F45”, after passing through the *LeftShift()*, the obtained bits are “9D73B04FD7F8FA2D”.
- *DNAReplication()* is applied on half of the sequence, which means the rest of the bits are unchanged after this iteration. The bits subjected to DNA replication have changed entirely, for example, if the STATE for the DNA layer is “9D73B04FD7F8FA2D”, from the preceding phase and after the replication process, the output is “9D73B04F**280705D2**”. The DNA replication process increases the diffusion and not the complexity [51,56], as it only required 16 new GEs for implementation on hardware.
- The *RightShift()* of nine bits improves the original sequence by breaking the already present bit sequence. If the value of STATE for the *RightShift()* is “9D73B04F280705D2”, then the sequence after the *RightShift()* will be “E94EB9D827940382”, which is entirely different from the previous round bits. The permuted bits from Figure 13 are, when subjected to original pLayer(), the resultant matrix is presented as Figure 14, where the first cell $\left[S_{0,58,0}\right]$ indicates a transition of bit zero with bit 58; however, in the original design, this transition never happened. Similarly, for second cell $\left[S_{1,10,16}\right]$, indicate the initial transition of bit 1 to the 10th position using new layers followed by the original pLayer() with the transition of this 10th bit to the 16th location.
- The reduced round concept is inspired by PRESENT’s improvements from [46,63] for low-power devices. They validated their improved design using the avalanche effect, throughput, and execution time. DNA-PRESENT has also observed 17 rounds and follows the same evaluation parameters for a fair comparison. Moreover, differential cryptanalysis is also used to establish confidence for the minimum number of rounds of DNA-PRESENT.

### 3.3. Implementation Environment and Test Vectors

The system specifications for implementing and evaluating DNA-PRESENT include an Intel(R) Core(TM) i5-8250U CPU running at 1.60 GHz, with 4 cores and 8 logical processors. These hardware details provide a suitable baseline for testing the algorithm’s performance in a practical, mid-level computational environment. The test vectors, as shown in Table 2, are presented in hexadecimal notation, covering plaintext, key, and ciphertext to facilitate clear understanding and reproducibility. This format allows precise verification of the implemented algorithm, as users can replicate the setup and validate results by comparing the generated ciphertext with expected outputs for given plaintext–key pairs.

To ensure reliability, each test was repeated multiple times and the results were averaged. This repetition aimed to minimize any fluctuations due to system processes and establish a consistent performance profile. Moreover, this repeated testing helped confirm that the observed performance improvements were not anomalies and remained consistent under varying operational conditions, simulating realistic usage scenarios. This methodology provides a rigorous framework for evaluating DNA-PRESENT, ensuring that the results reported are accurate and reproducible across similar setups.

## 4. Security, Cost, and Performance Analysis

**Figure 15:**
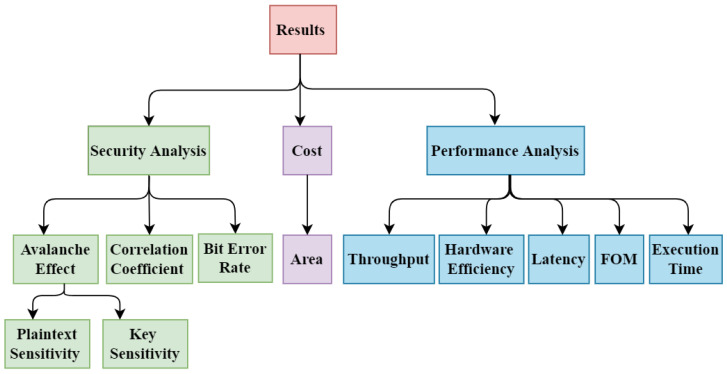
Categorization of tests for analysis.

The improvements in DNA-PRESENT were evaluated on each of the three cores of the triangle shown in Figure 1—security, cost, and performance—as depicted in Figure 15. These three categories evaluated the algorithm using specific tests and subtests. The datasets for evaluations are adopted from the literature [37,40,64,65,66], including Low-Density LD (all 0’s with at most two 1 bits), High-Density HD (all 1’s with at most two 0 bits), and random bits. In this experimental analysis, LDK, HDK, and RK represent Low-Density Key, High-Density Key, and Random Key, respectively, with a 128-bit key length. Meanwhile, LDP, HDP, and RP represent Low-Density Plaintext, High-Density Plaintext, and Random Plaintext, respectively, with 64-bit plaintext length. The experimental environment is kept the same for both algorithms for a fair comparison. Accordingly, the acquired results substantiate the robustness of the proposed methodology.

### 4.1. Security Analysis

Security is a primary requirement of any cryptographic algorithm. Confusion is a property that an encryption algorithm must possess to provide a complex relationship between plaintext and the ciphertext produced. Diffusion is when a slight change in plaintext disperses over the entire ciphertext [14,16,17,18,19,20,21,22,67]. Both confusion and diffusion are essential elements of a cryptographic algorithm to ensure optimal security [45]. Three experiments are conducted to evaluate these properties of the algorithms, including the avalanche effect, bit error rate, and correlation coefficient test. This evaluation methodology proves to be highly effective in assessing the security strength of lightweight block ciphers and can subsequently be adapted for other cryptographic primitives.

**Figure 16:**
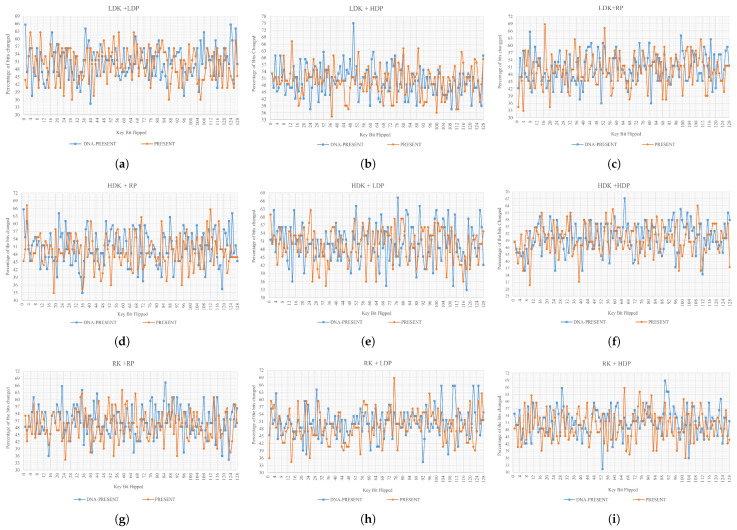
Graphical representation of key sensitivity test results (**a**–**i**).

**Figure 17:**
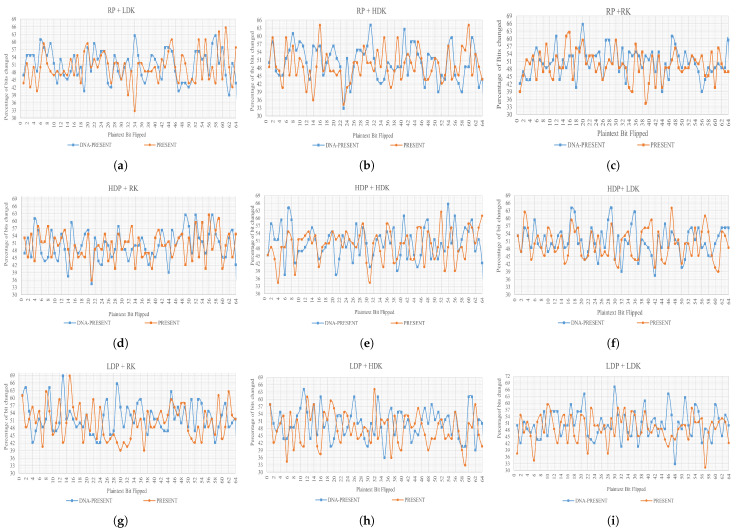
Graphical representation of plaintext sensitivity test results (**a**–**i**).

**Figure 18:**
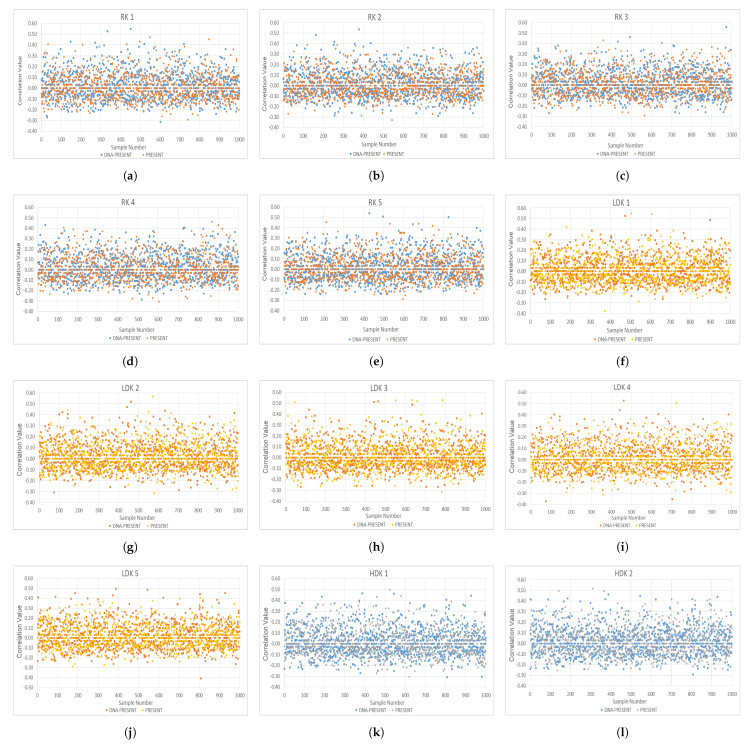
Graphicalrepresentation of correlation coefficient test results (**a**–**l**).

**Figure 19:**
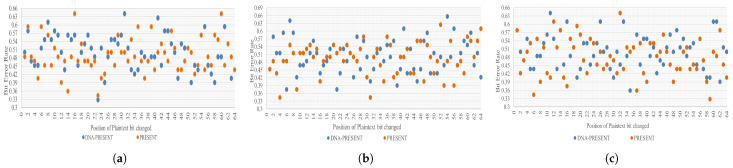
Graphical representation of bit error rate test results (**a**–**c**).

1. **Avalanche Effect (AE)**: The avalanche effect (AE) is one of the significant characteristics of cryptographic algorithms. It assesses the diffusion property of the algorithm where one bit in a key or in plaintext is changed to observe the bit difference in ciphertext (Equation (2)). In Equation (2), “x” denotes the length of the encrypted block, which is 64 bits for both algorithms, followed by “$c_{i}$” and “$p_{i}$”, which are the *i*th positioned ciphertext and plaintext bits. Later, in Equation (3), a percentage of the bits is calculated for a fair comparison. The AE value should be ≥50% to ensure perfect diffusion [67,68,69].
  (2) $$AE=\frac{1}{x}\sum_{\left(i=1\right)}^{x}\left|c_{i}-p_{i}\right|$$
  (3) Percentageofbitschanged=(AE)×100
  The test has been applied to both algorithms from two different perspectives, key sensitivity and plaintext sensitivity, and the datasets are given below in Table 3 and Table 4. The key sensitivity test will involve changing the value of the secret key, while the plaintext sensitivity test will require modifications to the plaintext bits. A perfect combination of each key against each plaintext has been created, where no key or plaintext combination has been ruled out in the evaluation. The exact numbers of secret keys, plaintext, derived ciphertext blocks, and obtained bits are presented in their respective columns. Derived blocks are directly proportional to the number of secret keys and plaintext for key and plaintext sensitivity. The derived bits are the ciphertext bits, which, for both PRESENT and DNA-PRESENT, are 64 bits (hence, derived blocks × 64 = derived bits).
  - **Key Sensitivity**: The size of the secret key is a major concern in symmetric encryption systems, and according to NIST, the key length should be ≥128 bits to ensure a basic defense against a brute force attack [70,71], which is met by DNA-PRESENT. Moreover, a well-designed algorithm should possess a desirable property of extreme sensitivity towards the bit change, where a small difference in the secret key can significantly affect the ciphertext even though the plaintext is kept constant [68]. Table 3 is used as the input for datasets while using Equations (2) and (3) to perform key sensitivity tests. The results are summarized in Table 5. A total of 1152 observations are derived and categorized into five different categories for a comprehensive review.
    The DNA-PRESENT has no observations in the ≤30% category. A 50% bit change is considered the ideal AE; for DNA-PRESENT, this value has increased to 10.33% from 9.81%. A perfect value with >50% AE has 4.6% more observations for DNA-PRESENT, indicating an evident increase in AE using DNA-PRESENT. The key sensitivity observed values are graphically presented in Figure 16a–i, following the same order of datasets as presented in Table 3, and exact observed values are plotted, respectively. These graphs endorse that DNA-PRESENT is more sensitive to key changes compared to PRESENT. It can be seen from the graphs that only the correct key can provide the exact plaintext if the secret key is a difference of even one bit; still, the ciphertext turns out to be very different.
  - **Plaintext Sensitivity**: This test evaluates the change in ciphertext while keeping the key constant and subjecting the plaintext to bit alterations, using the datasets provided in Table 4. Equations (2) and (3) were used along with 576 different plaintexts, while keeping the key constant, and the observations are summarized in Table 6. The observations are categorized similarly to key sensitivity analysis; here, a decrease of 1.04% in >30≤40% was achieved using DNA-PRESENT, and an increase of 3.65% was observed for >50% category. Following this table are the detailed graphs of each dataset in Figure 17a–i, depicting exact observed values in each dataset, and it can be seen that DNA-PRESENT achieves a higher bit difference in ciphertext when plaintext bits are toggled. Hence, the attacker can only intercept the plaintext if they have the exact ciphertext; otherwise, even one bit can cause a lot of difference.
2. **Correlation Coefficient**: A linear correlation between plaintext and ciphertext is assessed through the correlation coefficient test [72]. This test is a measure of confusion being introduced in the ciphertext. In a ciphertext-only attack, the attacker attempts to establish a relationship with the plaintext by obtaining the ciphertext. Each ciphertext is tested against its original plaintext, initially using the avalanche effect (Equation (2)) and later the correlation coefficient calculation using Equation (4), as given below. Here, AE is the avalanche effect; *ith* plaintext and the ciphertext bits are represented as $p_{i}$ and $c_{i}$, respectively [73]. The value “R” derived from the test spans from −1 to +1, indicating the correlation between plaintext and ciphertext, summarized in Table 7. The value “R = 0” is ideal, as plaintext and ciphertext are independent of each other, and an attacker cannot establish any relationship between them by analyzing any given one. On a larger scale, the absolute values reveal the strength of the relationship—where the smaller the number, the weaker the relationship—aligning with the intended goal of cryptographic algorithms. To validate this relationship, a dataset of 1000 random plaintexts was subjected to three key categories, ranging from RK, LDK, to HDK.
  For the test, a total of 15,000 values were observed, and the distribution of observations across each category is summarized in Table 8. For all other scenarios, the values are very close; however, there is an increase of 0.22% for R = 0 using DNA-PRESENT, highlighting a larger number of observations where no relationship can be established between the plaintext and ciphertext by the attacker. There is a decrease in weak negative relations with 0.5. These findings suggest that the enhanced algorithm has successfully reduced the linear relationship in fewer rounds, thereby concealing more information about the plaintext when presented with the ciphertext. This also endorses that the improved and original PRESENT are secure against ciphertext attacks. The exact values are depicted in graphs as Figure 18a–e for RK, Figure 18f–j for LDK, and Figure 18k–o for HDK values.
  (4) $$R=\frac{\sum_{\left(i=1\right)}^{s}\left(p_{i}-AE\right)\left(c_{i}-AE\right)}{\sqrt{\sum_{\left(i=1\right)}^{s}{\left(p_{i}-AE\right)}^{2}}\sqrt{\sum_{\left(i=1\right)}^{s}{\left(c_{i}-AE\right)}^{2}}}$$
3. **Bit Error Rate**: The bit error rate (BER) test measures the bit difference in ciphertext when subjected to bit change in plaintext. The error transmitted in the ciphertext corresponds to the total number of bits that change when one bit in the plaintext is modified [73,74,75]. The bit error should be 50% or more bits in a good algorithm. This test measures the relationship between plaintext and ciphertext. As a mathematical expression, this BER can be expressed as Equation (5):
  (5) $$BER=\frac{Number\;of\;cipher\;bit\;difference}{Total\;number\;of\;ciphertext\;bits}$$
  Three different datasets of plaintext are compared for BER against HDK. Individual values of each dataset are presented in Table 9. On average, the original technique has a bit difference of 31 bits, whereas DNA-PRESENT consistently achieved a 32-bit difference, which accurately reflects a 50% error rate. A pictorial representation of all the observations for the three scenarios mentioned above is presented in Figure 19. The number of plaintext bits modified for each observation is indicated on the X-axis, and the Y-axis contains the bit error rate (BER) values.

### 4.2. Cost

The implementation cost is directly influenced by the design of an algorithm. The area for hardware implementation is measured in Gate Equivalence (GE), reflecting both computational complexity and the chip area required (cost) for its implementation. Technically, it is the ratio between the layout area (measured in $\mu m^{2}$) and the NAND 2 gate area [33]. GE is a crucial element in lightweight cryptography and should have a small value. According to the authors [8,76], only 3000 gates are dedicated for security algorithms from a total of 1000–10,000 gates in resource-constrained devices. This implementation’s GE is calculated using the base paper’s fundamentals [25]. As indicated in Table 10, a significant proportion of the gates in both algorithms is accounted for by the key schedule algorithm (KSA). Notably, the recently incorporated layers only add 16 new gates, representing a negligible cost when weighed against the security improvements. The total number of gates required by PRESENT-128 is 1886.25, which increased to 2518.85 GEs for DNA-PRESENT; nonetheless, a trade-off consistently exists between achieving improved security and maintaining lower costs. Although this number is higher than the original PRESENT block cipher, the algorithm is still a lightweight solution aiming to be implemented using less than 3000 GE’s [8,33,76]. It is pertinent to mention that the discussion of the DNA-based solution is primarily due to its simplicity, with the gate count required for the DNA-replication operation being only 16 GEs, which underscores its lightweight nature. However, it is important to note that this solution does not meet the criteria for ultra-lightweight algorithms, which may present potential limitations for certain IoT environments.

### 4.3. Performance Analysis

A performance analysis of DNA-PRESENT against other lightweight block ciphers is performed and summarized in this section. This study specifically focuses on block ciphers, with both the benchmarks and proposed algorithm belonging to this category. While there are many other prominent techniques, such as stream ciphers, a direct comparison with these would not have been entirely appropriate and may have introduced confusion due to the fundamental differences. Therefore, the selected algorithms range from LED, RECTANGLE, PRESENT, DNA-PRESENT, PUFFIN, mCrypton, I-PRESENT, NOEKEON, PRINCE, and ICEBERG to AES. These algorithms have three things in common: SPN structure, 128-bit key size, and applicability to resource-constrained devices. These algorithms are compared against DNA-PRESENT and summarized in Table 11. These algorithms are compared for block size, key size, and number of rounds, followed by the cost comparison as GE. The hardware performance in the form of latency, throughput, hardware efficiency, and figure of merit are also used as evaluation parameters and are explained as follows:

1. **Latency**: In IoT, resource-constrained devices need to be responsive around the clock and do not require a time lag; hence, latency becomes an important evaluation criterion for block ciphers. The time involved in encrypting a single block of plaintext during the encryption process is termed latency, [12,33,77,78], mathematically expressed as Equation (6).
  (6) $$L=k\times t_{cycles}$$
  *L* is the latency, the variable *k* denotes the number of clock cycles required for computing a single block of plaintext and its corresponding ciphertext, and $t_{cycles}$ is the time required for one cycle. ICEBERG has the lowest latency with 16 Cycles/block, followed by DNA-PRESENT with 17 Cycles/block.
2. **Throughput (TP)**: The number of bits produced per second at a designated frequency in the encryption–decryption process is referred to as throughput [33]. This throughput is always calculated at a specified frequency, and it is 100 kHz for hardware implementation. Mathematically, throughput can be expressed as Equation (7):
  (7) $$Throughput=\frac{Block\;Size\times Frequency}{Number\;of\;cycles\;per\;block}$$
  The throughput is measured in Kb/s, and ICEBERG achieved the highest value with 400 Kb/s followed by DNA-PRESENT with 376.47 Kb/s.
3. **Hardware Efficiency (HE)**: Hardware efficiency is a critical parameter for evaluating algorithms as it represents the ratio of performance to the incurred cost [33]. The performance is the value obtained from the throughput, whereas the cost is the GE for hardware implementation. The highest HE is observed for PRINCE with 152.77 Kbps/KGE, followed by DNA-PRESENT with 149.45 Kbps/KGE.
4. **Figure of Merit (FoM)**: The performance of a block cipher is subjected to an ultimate evaluation matrix, i.e., figure of merit (FoM) presented by [79]. It was introduced to overcome the weaknesses in the existing hardware evaluation matrices by finding the optimal cost performance evaluation. Mathematically, it can be expressed as Equation (8):
  (8) $$FoM=\frac{T}{A^{2}}$$
  where *T* is the throughput and *A* is the area of implementation measured as GE. This FoM helps in algorithm decisions based on the cost and performance trade-offs during algorithm selection. Initially, the highest value in FoM was observed for the PRESENT block cipher with a value of 56.23; now, the highest achieved value is by DNA-PRESENT at 59.32. This indicates that DNA-PRESENT is now observing the perfect cost–performance balance, which was previously achieved by PRESENT.
  These 10 lightweight solutions are sorted based on their relative area concerning DNA-PRESENT. The last column of Table 11 has the relative size of these lightweight block ciphers compared to DNA-PRESENT. The LED, RECTANGLE, and PRESENT block ciphers have relatively fewer area requirements than DNA-PRESENT, but six other algorithms have a higher cost. This observation indicates that DNA-PRESENT does not have the lowest number of gates but still has relatively fewer gates than other similar block ciphers, as it is ranked 4th among the 10 block ciphers under consideration. This endorses that the improved cost is still very competitive compared to other lightweight solutions for the security gain achieved.
5. **Execution Time**: Execution time is a measure to identify the time difference as a trade-off to achieve security. Execution time is calculated from three perspectives—encryption, decryption, and overall execution of the complete algorithm [46]. It is performed on 100 plaintexts for both PRESENT and DNA-PRESENT, and the average time is included as a measure. The encryption time for PRESENT is 0.141 s, and it is 0.1201 s using DNA-PRESENT. At the same time, the decryption time is 0.1751 s and 0.1132 s for PRESENT and DNA-PRESENT, respectively. For one plaintext, the total execution time is 0.3161 s and 0.2333 s for PRESENT and DNA-PRESENT, respectively. It can be seen that the execution time of DNA-PRESENT decreased compared to PRESENT because of the lesser number of rounds.

## 5. Differential Cryptanalysis of DNA-PRESENT

Differential cryptanalysis, introduced by Biham and Shamir [80], is one of the most effective techniques for assessing the security strength of a cipher. It is a chosen plaintext–ciphertext attack, where the attacker can observe the encrypted output after selecting specific plaintexts as inputs to the cipher. To perform differential cryptanalysis, the attacker uses a pair of plaintexts that differ by a constant value. A differential path or trail represents a sequence of differences across multiple rounds of encryption. These paths are primarily established for the cipher’s nonlinear component, which, in the PRESENT block cipher, is the s-box. While finding a differential path, there are multiple output differences against a particular input difference for s-box. Therefore, a difference distribution table of the s-box serves as a starting point for attackers, giving a certain output probability over input, as presented in [81]. The differential paths were only found until round 8, as shown in Table 12, and the cipher is secured for the full number of rounds. The maximum probability of difference propagation is observed at the 8th round of $2^{-79}$, lower than $2^{-63}$, and presented in detail for each round from round 1 to round 8. As a result, it becomes impossible to build a successful differential distinguisher for more than eight rounds for the DNA-PRESENT algorithm. It is good to double the number of rounds to obtain complete diffusion as it will provide a larger security margin and confidence interval [81]. According to [81], the number of rounds should be odd as it is a common practice, and there are 16 rounds of encryption with an additional round of key whitening, so the total number of rounds is 17. Hence, full-round DNA-PRESENT is enough to resist the differential attack. Calculating the optimal number of rounds for our proposed design involved using a differential cryptanalysis approach to find the differential trails. It is common to perform differential trails to the secure bound on the number of rounds [13,29,81,82].

## 6. Comparison Analysis

The validation of encryption algorithms typically hinges upon three critical factors: security, cost, and performance. In resource-constrained environments, where lightweight cryptographic solutions are essential, it is imperative that any proposed design not only enhances security but also maintains efficiency with minimal resource overhead. DNA-PRESENT, the proposed variant of the PRESENT block cipher, has undergone rigorous evaluation against six major cryptographic criteria. These evaluations were conducted in direct comparison with the base algorithm, PRESENT, and the results unequivocally demonstrate that DNA-PRESENT achieves superior performance in key security metrics, including confusion and diffusion, with a reduced number of rounds.

In terms of cryptographic strength, DNA-PRESENT outperforms PRESENT in several critical areas—notably, the avalanche effect, key sensitivity, plaintext sensitivity, bit error rate, and resilience against differential trails. These improvements underscore the robustness of the proposed scheme in enhancing the security of lightweight cryptographic solutions without compromising efficiency.

While it is acknowledged that the implementation cost of DNA-PRESENT slightly increased due to the integration of additional operations, the design remains well within the lightweight cryptography domain. The gate count was carefully managed to strike an optimal balance between enhanced security and minimal hardware overhead, ensuring that DNA-PRESENT adheres to the stringent requirements of lightweight encryption algorithms. Importantly, the execution time of DNA-PRESENT was also reduced when compared to the original PRESENT cipher. This reduction in execution time, despite the introduction of new operations, can be attributed to the decrease in the number of rounds required, resulting in faster encryption and decryption processes.

Overall, DNA-PRESENT represents a significant advancement over PRESENT, offering a refined balance of security and efficiency. The trade-offs made in terms of implementation cost are offset by substantial improvements in both cryptographic strength and operational speed, making it a highly suitable candidate for secure communication in resource-limited environments.

## 7. Conclusions

This study sets out to design an improved PRESENT block cipher named DNA-PRESENT. The proposed enhancement introduced the left shift, right shift, and DNA replication processes to increase the original PRESENT’s diffusion, followed by a decrease in latency. DNA-PRESENT was evaluated against PRESENT for security, cost, and performance analysis using five specific tests. The experimental results confirm that DNA-PRESENT outperforms the original PRESENT in security analysis with an evident increase in the avalanche effect. An increase in cost was observed while achieving improved security in DNA-PRESENT. Performance analysis was performed against other state-of-the-art lightweight algorithms, indicating a significant decrease in latency and an increase in throughput. Moreover, the execution time for DNA-PRESENT also decreased to 0.2333 s from 0.3161 s, which makes it a fitting choice for deployment in resource-constrained environments. This extensive comparative analysis demonstrates the superiority of DNA-PRESENT against other state-of-the-art lightweight algorithms. It would be interesting to extend this study for PRESENT-80 in the future and to analyze the impact of these enhancements on security and performance. This research contributes to future studies on lightweight block ciphers, and their extensive comparison of cost, security, and performance, while standing out as a promising choice for resource-constrained devices.

While this study demonstrates the significant security and performance enhancements of DNA-PRESENT over the base algorithm, PRESENT, the potential vulnerabilities introduced by DNA-based cryptographic operations were not exhaustively explored in this work. Future research may focus on performing a comprehensive security analysis to assess any new attack surfaces that may arise from these operations. This exploration will further strengthen the robustness of DNA-PRESENT over a wide range of security scenarios. In addition, future research may include detailed security proofs through security protocol verification tools.

## Figures and Tables

**Table 1:** Substitution box of PRESENT block cipher.

X	0	1	2	3	4	5	6	7	8	9	A	B	C	D	E	F
S(X)	C	5	6	B	9	0	A	D	3	E	F	8	4	7	1	2

**Table 2:** Test vectors for DNA-PRESENT algorithm.

Plaintext	Secret Key	Ciphertext
0000000000000000	0000000000000000 0000000000000000	6BB502A27FE7DAC8
0000000000000000	FFFFFFFFFFFFFFFFF FFFFFFFFFFFFFFFF	3B88A4E8441280FE
0000000000000000	18546B83039E89EE1 D41DC9E5F9AC1751	7B4203198F79F29
FFFFFFFFFFFFFFFF	0000000000000000 0000000000000000	52F220F43D1C838D
FFFFFFFFFFFFFFFF	FFFFFFFFFFFFFFFF FFFFFFFFFFFFFFFF	541779374B18633C
FFFFFFFFFFFFFFFF	18546B83039E89EE D41DC9E5F9AC1751	80595BE55A422F7E
996ADBEEF0143262	0000000000000000 0000000000000000	AD1928EF9EC07B82
996ADBEEF0143262	FFFFFFFFFFFFFFFF FFFFFFFFFFFFFFFF	45BC53ED28EA9502
996ADBEEF0143262	18546B83039E89EE D41DC9E5F9AC1751	E77D539DF778FF91

**Table 3:** Input data categories for key sensitivity test.

Set No.	Data Category	Secret Key	Plaintext	Derived Blocks	Derived Bits
1	LDK + LDP	128 Specific	1	128	8192
2	LDK+ HDP	128 Specific	1	128	8192
3	LDK + RP	128 Specific	1	128	8192
4	HDK+ RP	128 Specific	1	128	8192
5	HDK + LDP	128 Specific	1	128	8192
6	HDK + HDP	128 Specific	1	128	8192
7	RK+ RP	128 Specific	1	128	8192
8	RK + LDP	128 Specific	1	128	8192
9	RK + HDP	128 Specific	1	128	8192
	Total	1152 Specific	9	1152	73,728

**Table 4:** Input data categories for plaintext sensitivity test.

Set No.	Data Category	Secret Key	Plaintext	Derived Blocks	Derived Bits
1	RP + LDK	1	64 Specific	64	4096
2	LDK+ HDP	1	64 Specific	64	4096
3	RP + RK	1	64 Specific	64	4096
4	HDP + RK	1	64 Specific	64	4096
5	HDP + HDK	1	64 Specific	64	4096
6	HDP + LDK	1	64 Specific	64	4096
7	LDP + RK	1	64 Specific	64	4096
8	LDP + HDK	1	64 Specific	64	4096
9	LDP + LDK	1	64 Specific	64	4096
	Total	9	576 Specific	576	36,864

**Table 5:** Key sensitivity analysis.

DNA-PRESENT					PRESENT					
Set No.	≤30%	>30 ≤ 40%	>40<50%	=50%	>50%	≤30%	>30 ≤ 40%	>40<50%	=50%	>50%
1	0	5	48	9	66	0	5	52	8	63
2	0	7	56	9	56	0	12	46	14	56
3	0	3	54	10	61	0	12	48	10	58
4	0	9	47	12	60	0	9	58	17	44
5	0	9	50	14	55	0	7	44	16	61
6	0	8	47	15	58	1	7	53	12	55
7	0	6	49	14	59	0	9	55	16	48
8	0	5	44	25	54	0	9	60	13	46
9	0	2	42	11	73	0	8	55	7	58
Avg	0	6	48.6	13.2	60.2	0.1	8.7	52.3	12.6	54.3

**Table 6:** Plaintext sensitivity analysis.

DNA-PRESENT					PRESENT					
Set No.	≤30%	>30 ≤ 40%	>40<50%	=50%	>50%	≤30%	>30 ≤ 40%	>40<50%	=50%	>50%
1	0	1	27	3	33	0	2	27	9	26
2	0	4	24	5	31	0	3	35	7	19
3	0	2	23	7	32	0	3	29	7	25
4	0	3	26	8	27	0	1	26	7	30
5	0	3	26	4	31	0	5	23	9	27
6	0	2	21	10	31	0	1	30	40	29
7	0	0	24	5	35	0	2	27	4	31
8	0	2	26	5	31	0	8	26	7	23
9	0	1	27	8	28	0	5	26	6	27
Avg	0	2	24.9	6.1	31	0	3.3	27.7	6.7	26.3

**Table 7:** Correlation coefficient test result indicators.

Sr. No	Condition	Result
1	{R = 0}	Strong positive linear relationship
2	{0<R≤0.3}	Weak positive linear relationship
3	{−0.3≤R<0}	Weak negative linear relationship
4	{0.3 < R < 0.7}	Moderate positive linear relationship
5	{−0.7<R<−0.3}	Moderate negative linear relationship
6	{0.7≤R<1}	Strong positive linear relationship
7	{−1<R≤−0.7}	Strong negative linear relationship
8	{R = 1}	Perfect positive linear relationship
9	{R = −1}	Perfect negative linear relationship

**Table 8:** Correlation coefficient test results.

DNA-PRESENT					PRESENT					
Key	{R = 0}	{0<R≤0.3}	{−0.3≤R<0}	{0.3 < R < 0.7}	{−0.7<R<−0.3}	{R = 0}	{0<R≤0.3}	{−0.3≤R<0}	{0.3 < R < 0.7}	{−0.7<R<−0.3}
RK1	93	463	420	23	1	104	414	465	17	0
RK2	94	441	446	19	0	90	429	469	12	0
RK3	85	463	432	20	0	99	459	428	14	0
RK4	126	442	410	22	0	104	421	454	21	0
RK5	102	442	440	16	0	106	449	427	18	0
LDK1	109	410	468	13	0	102	420	474	13	1
LDK2	101	442	430	26	1	115	448	419	17	1
LDK3	128	385	466	21	0	97	440	440	22	1
LDK4	94	457	427	20	2	106	428	450	15	1
LDK5	91	449	438	21	1	98	443	444	15	0
HDK1	109	417	449	24	1	110	402	464	24	0
HDK2	107	446	417	30	0	77	453	448	22	0
HDK3	96	415	472	16	1	96	478	405	21	0
HDK4	105	425	453	17	0	106	428	445	21	0
HDK5	96	443	438	23	0	93	434	453	19	1
Avg	102.4	436.0	440.4	20.7	0.5	100.2	436.4	445.7	18.1	0.3
%	10.24	43.60	44.04	2.07	0.05	10.02	43.64	44.57	1.81	0.03

**Table 9:** BER comparison of DNA-PRESENT and PRESENT.

DNA-PRESENT			PRESENT	
**Input**	**Average Different Bits**	**Average BER**	**Average Different Bits**	**Average BER**
RP	32.125	0.501953125	31.28125	0.488769531
HDP	32.140625	0.502197266	31.609375	0.493896484
LDP	32.140625	0.502197266	30.65625	0.479003906
Avg	32.135	0.50211588	31.1822	0.4872233

**Table 10:** Cost requirements for DNA-PRESENT and PRESENT.

DNA-PRESENT			PRESENT	
Module	GE	%age	GE	%age
Data Sate	384.39	15.26%	384.39	20.38%
S-Layer	448.45	17.80%	448.45	23.77%
Left-shift	0	0	N/A	N/A
DNA Replication	16	0.64%	N/A	N/A
Right Shift	0	0	N/A	N/A
P-Layer	0	0	0	0
Counter.State	28.36	1.13	28.36	1.50%
Counter.Combinatorial	12.35	0.49	12.35	0.65%
KSA	1625.65	64.53%	1009.03	53.50%
Other	3.67	0.15	3.67	0.19%
Total	2518.85	100%	1886.25	100%

**Table 11:** Cost, latency, throughput, hardware efficiency, FOM, and relative size of block ciphers with SPN structure.

Algorithm	Block	Key	Rounds	Tech	Cost	Latency	TP	HE	FoM	Size with Respect to DNA-PRESENT
Unit	Bits	Bits		µm	GE	Cycles/Block	Kb/s	Kbps/KGE		
Better Is			Lower		Lower	Lower	Higher	Higher	Higher	Lower
LED [12,27]	64	128	48	0.18	1265	1872	3.4	2.68	2.12	0.50
RECTANGLE [28]	64	128	25	0.13	1787	246	26	137.66	8.14	0.71
PRESENT [25]	64	128	31	0.18	1886	32	200	106.04	56.23	0.75
DNA-PRESENT	64	128	17	0.18	2519	17	376.47	149.45	59.32	
PUFFIN [12,26]	64	128	32	0.18	2577	32	200	77.6	30.12	1.02
mCrypton [12]	64	128	12	0.18	2760	190	33.51	12.14	4.40	1.10
I-PRESENT [29]	64	128	30	0.18	2783	30	213.33	76.65	27.54	1.1
NOEKEON [23]	128	128	16	0.18	2862	3720	3.44	1.2	0.42	1.38
PRINCE [12]	64	128	11	0.13	3491	533.3	12	152.77	0.98	1.47
ICEBERG [24]	64	128	16	0.18	5817	16	400	68.76	11.82	2.31
AES [13]	128	128	10	0.13	11,031	226	48	4.35	0.39	4.38

**Table 12:** Differential trails and number of active s-boxes.

Round Number	Probability	Active s-Boxes
1	$2^{-2}$	2
2	$2^{-6}$	4
3	$2^{-18}$	4
4	$2^{-30}$	3
5	$2^{-38}$	3
6	$2^{-47}$	6
7	$2^{-61}$	6
8	$2^{-79}$	5
Total		33

## Data Availability

The original contributions presented in the study are included in the article, further inquiries can be directed to the corresponding authors.

## Abbreviations

The following abbreviations are used in this manuscript:

IoT	Internet of Things
DNA	Deoxyribonucleic Acid
BER	Bit Error Rate
RFID	Radio Frequency Identification
LWC	Lightweight Cryptography
GE	Gate Equivalance
SPN	Substitution Permutation Network
s-box/sBoxLayer	Substitution box
p-box/pLayer	Permutation box
AES	Advance Encryption Standard
ISO	International Organization for Standardization
IEC	International Electrotechnical Commission
CDMB	Central Dogma of Molecular Biology
LD	Low Density
HD	High Density
AE	Avalanche Effect
KSA	Key Schedule Algorithm
TP	Throughput
HE	Hardware Efficiency
FoM	Figure of Merit
